# An experimental study of gender and cultural differences in hue preference

**DOI:** 10.3389/fpsyg.2015.00030

**Published:** 2015-01-30

**Authors:** Abdulrahman S. Al-Rasheed

**Affiliations:** Department of Psychology, King Saud University, Riyadh, Saudi Arabia

**Keywords:** color preference, hue preference, gender differences, culture differences

## Abstract

This paper investigates the influence of both gender and culture on color preference. Inspection of previous studies of color preference reveals that many of these studies have poor control over the colors that are shown—the chromatic co-ordinates of colors are either not noted or the illuminant that colors are shown under is not controlled. This means that conclusions about color preference are made using subjective terms for hue with little knowledge about the precise colors that were shown. However, recently, a new quantitative approach to investigating color preference has been proposed, where there is no need to summarize color preference using subjective terms for hue (Hurlbert and Ling, 2007; Ling and Hurlbert, 2007). This approach aims to quantitatively summarize hue preference in terms of weights on the two channels or “cardinal axes” underlying color vision. Here I further extend Hurlbert and Ling’s (2007) approach to investigating color preference, by replicating their study but with Arabic and English participants, and to answer several questions: First, are there cultural differences in the shape of the overall preference curve for English and Arabic participants? Second, are there gender differences in the shape of the overall preference curve for English and Arabic participants? Thirty eight British and 71 Saudi Arabian (Arabic) participants were compared. Results revealed that Arabic and English preference curves were found to differ, yet there was greater similarity for Arabic and English males than Arabic and English females. There was also a sex difference that was present for both Arabic and English participants. The male curve is fairly similar for both samples: peak-preference is in the blue-green region, and a preference minimum is in the red-pink/purple region. For Arabic females the preference peak appears to be in the red-pink region, whilst for English females it is shifted toward purple/blue-green.

## INTRODUCTION

Color usually evokes an aesthetic, expressed, for instance, in terms of preference for some colors over others. According to Chandler (1934), studies of color preference date back to at least 1800, addressing questions such as: do people tend to prefer the same colors; do the sexes differ in patterns of preference? Embedded in both questions, is the issue of what determines preference; to what extent is it Universal, or in contrast, to what extent is it peculiar to the individual? If there are Universal patterns, are these determined by our genes, or by common experience? If experience is important, then there could be consistent cultural differences as people from the same culture are more likely to have similar experiences than people from different cultures.

Early studies were marred by lack of control over the specification of the colors and of the illuminant they were viewed under. However, half a century ago it was established that, preferences were highest for the blue-green region and lowest for the yellow and yellow-green regions (Guilford and Smith, 1959). Moreover, the preference order reported by Eysenck (1941)—blue, red, green, purple, orange, and yellow—has generally been supported by more recent studies (see Ling et al., 2006 for a summary). Note however, the majority of these studies were conducted on Western European or American informants, and the consensus does not extend to the issue of cross-cultural variation.

### GENDER DIFFERENCES IN COLOR PREFERENCE

The debate about whether gender plays a role in hue preference has raged since as long ago as 1800 (Chandler, 1934). Eysenck (1941) found gender differences only for orange and yellow, while Granger (1955) concluded from his controlled ranking study of more than 400 Munsell colors covering the entire color solid, that there was no evidence of any marked difference between the preference ranking of men and women. A child hue preference study by Zentner (2001) tested 127 Swiss preschool children (mean age = 54 months) with nine color patches (black, light blue, dark blue, brown, light green, dark green, pink, red, and yellow), and children handed the experimenter the colors in order of their preference. Results showed no significant effect of gender on color preference. Also, studies by Child et al. (1968), Camgoz et al. (2002), Ou et al. (2003), and Rosenbloom (2006) have shown no significant gender difference in hue preference.

On the other hand, other studies report substantial gender differences, and have shown that males and females differ when it comes to their favorite colors. An early major finding was that females showed a greater preference for warm colors than males and males showed a greater preference for cool colors than females (Helson and Lansford, 1970). McManus et al. (1981) have also concluded from a controlled paired comparison task, that males and females differ significantly in their hue preference, as females showed a greater preference for red and less preference for yellow compared to males. Further evidence comes from a developmental study by Burkitt et al. (2003) who tested the color preference of 330 UK children, aged between 4 and 11 years old. Children were asked to point to their preferred color from a set of 10 colors (black, blue, brown, green, orange, pink, purple, red, white, and yellow), and continued pointing until all colors were chosen. It was found that girls significantly preferred pink, purple, and red more than boys. In contrast boys showed a greater preference than girls for black, blue, brown, green, and white. More recent work by Ling and Hurlbert (2007), tested 94 males and females from China and England, aged between 20 and 26, and they were asked as quickly as possible to choose their preferred color from each of a series of paired colored rectangles. The findings from this study showed that females prefer reddish hues and dislike greenish-yellowish hues significantly more than males. These sex differences in hue preference could be due to culture (Langenbeck, 1913). For example, Paoletti (1983, 1987, 1997) documented that sex difference in color preference is culturally influenced and noted that general acceptance of pink for girls and blue for boys was inverted since 1920 in the North American culture. Cultural color stereotypes which influence color preference are found in Eichstedt’s (1997) study where the awareness of gender incongruity led 24 months old to look longer at a pink hue than blue hue when it was preceded by a male than a female voice. Sex differences in color preference could also be due to biological factors (Humphrey, 1976) or due to sex differences in the evolution of color vision (e.g., Hurlbert and Ling, 2007). This issue is returned to later on.

### CULTURAL DIFFERENCES IN COLOR PREFERENCE

Colors have different meanings in different cultures. For instant, red symbolizes good luck in China, Denmark, and Argentina, while it means bad luck in Germany, Nigeria, and Chad (Schmitt, 1995; Neal et al., 2002). White is a color of happiness and purity in the USA, Australia, and New Zealand, but symbolizes death in East Asia (Ricks, 1983; Neal et al., 2002). Green represents envy in the USA and Belgium, while in Malaysia it represents danger or disease (Ricks, 1983; Hupka et al., 1997). This variation in the symbolism of color could lead to variation on color preference between cultures. Choungourian (1969) compared 148 American and Lebanese children age between 5 and 10 using eight colored stimuli (red, orange, yellow, yellow-green, green, turquoise, blue, and purple) and the eight colors were paired against each other. Color preference varied between the two samples, American children showed a significant preference for red and lack of preference for green, whereas Lebanese children showed preference for blue and lack of preference for green, and their top and bottom of preference did not differ significantly from each other.

A previous comparative adult study by Choungourian (1968) which compared 160 male and female American, Lebanese, Iranian, and Kuwaiti university students, also found cultural variation in color preference. By using the same stimuli and method, results showed variation in the over-all order of color preference across the four cultures. Saito (1994) compared Japanese, Korean, and Taipei samples. Participants were asked to select from a color chart the three colors they preferred most and the three they disliked the most and, to give their reasons for their choices. Results of these studies showed that although a high preference for white was common to all three samples, each sample had a specific preference for colors not shown by the others. Saito (1996) also expanded their study using the same stimuli and procedure but testing 175 Japanese, 158 Chinese, and 157 Indonesian participants. The finding of this study confirmed the conclusion of the previous study that culture influenced color preference. More recently, Ling et al. (2006) and Hurlbert and Ling (2007) reported that cultural factors played a role in differences in the color preference of English and Chinese participants. The Chinese sample had a stronger preference for reddish hues than the English sample, and it was argued that this variation is due to a red symbolizing good luck in the Chinese culture.

### AIMS OF THE STUDY

The main aim of this study was to extend Ling and Hurlbert (2007) and Hurlbert and Ling’s (2007) work, by replicating their study with Arabic participants. One aim was to investigate whether the sex difference found for English and Chinese samples would also be found in a Saudi Arabian Arab sample. If so, this would be support for sex difference in the weighting of this biological component being universal and possibly linked to evolutionary processes. In addition, the study aimed to contribute to the debate about how color preference is constrained, whilst also testing whether differential weighting of the cardinal axes of color vision could explain variation in color preference for cultures other than English and Chinese.

## METHOD

### PARTICIPANTS

Seventy-one native Arabic-speaking undergraduates from King Saud University in Riyadh and 38 native English-speaking undergraduates from Surrey University participated in this experiment. All their ages ranged from 18 to 29 years. For the Arabic sample, there were 32 males with a mean age of 21.35 years (SD = 1.47), and 36 females with a mean age of 20.28 years (SD = 0.54). For the English sample, there were 17 males with a mean age of 21.35 years (SD = 3.32), and 31 females with a mean age of 19.32 years (SD = 2.06). All participants had normal red-green color vision as assessed by Ishihara’s Tests for Color Vision Deficiency (Ishihara, 1987). Most of the participants participated for course credit and a few volunteered. No participant was aware of the predictions of the experiment at the time of testing.

### STIMULI AND APPARATUS

As in Ling and Hurlbert (2007), there were eight stimuli that varied only in hue angle (saturation = 0.5 and lightness = 80 in CIE Lu’v’ HSL space). Table 1 shows the coordinates in CIE (1931) Y,x,y space for the eight stimuli, against the uniform gray background (Y = 50 cd/m^2^, x = 0.321, y = 0.337). A Cambridge Research Instruments ColorCal was used to obtain CIE co-ordinates. The color stimuli were displayed on a calibrated 17 inch CRT Sony Trinitron monitor.

**Table 1:** **CIE (1931), Y,x,y co-ordinates of the eight stimuli and the hue angle and the hue labels for all the stimuli: YR, yellow-red; R, red; RP, red-pink; P, pink; BG, blue-green; G, green; GY, green-yellow**.

**Stimulus**	**Y**	**x**	**y**	**Hue angle**	**Hue label**
1	28.29	0.376	0.342	1.29	YR
2	28.28	0.362	0.316	1.69	R
3	28.32	0.346	0.299	2.03	RP
4	28.29	0.295	0.278	3.01	P
5	28.32	0.264	0.332	4.42	BG
6	28.32	0.274	0.360	4.84	G
7	28.29	0.291	0.382	5.17	G
8	28.32	0.353	0.410	6.15	GY

Stimuli were constant saturation (S = 0.5) and constant lightness (L = 80).

### DESIGN

The stimuli were presented as pairs of rectangular patches (3 cm × 2 cm) above and below central fixation (4 cm between the two patches) on the gray background. Each possible pair was shown twice, in random order, with the position of each color reversed on the second occurrence.

### PROCEDURE

The experiment was conducted in a dark room. Participants were seated at a distance of 57 cm from the monitor, at eye-level to the center of the monitor, with head restrained using a chin rest. Participants were instructed to move the cursor as quickly as possible to select their preferred color in each pair. The next pair appeared immediately after each response until the end of task. Participants were told that, there was no time limit for their responding, and not to think about possible uses of the color.

## RESULTS

**FIGURE 1:**
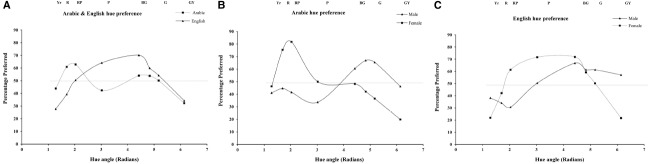
**The hue preference curve (% preferred for each stimulus) for males and females, for the Arabic and English samples.** The Munsell hue labels for the stimulus range are given. The dashed line at 50% indicates no preference.

The number of times each hue chosen as the preferred color across all the 56 trials was calculated for each participant and the mean across participants percentage preference scores for each color are shown in Figure 1A which gives the hue preference curves for the Arabic and English samples. It can be seen that there appear to be differences in the preference curves for Arabic and English samples. For the Arabic sample, the preference peak is in the red-pink region, with a preference minimum in the green-yellow region. For the English sample, peak preference is in the purple/blue-green region, with a preference minimum in the yellow-red region. The preference peak differs across the two samples, but the samples share a dislike of yellowish colors.

Figures 1B,C illustrate the hue preference curves by plotting the percentage preferred, for the eight stimuli for males vs. females for each of the Arabic and English samples. Figure 1B gives male and female preference curves for the Arabic sample while Figure 1C gives male and female preference curves for the English sample. From Figure 1, it can be seen that there appear to be sex differences in color preference for both groups. The male curve is fairly similar for both samples: peak-preference is in the blue-green region, and a preference minimum is in the red-pink/purple region. For Arabic females the preference peak appears to be in the red-pink region, whilst for English females it is shifted toward purple/blue-green. Both Arabic and English females however, appear to dislike the green-yellow hue.

**FIGURE 2:**
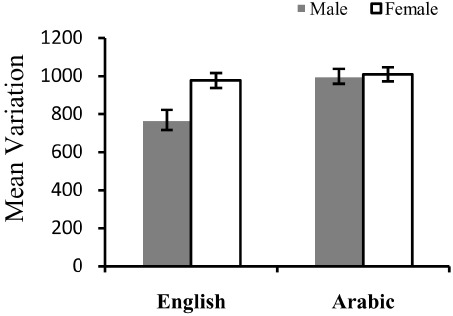
**Mean variation (±1 SE) for males and females of Arabic and English samples**.

The variance of each participant’s preference curve was calculated. This represents how strong the variation in preference there is across the hues tested—smaller numbers indicate less variation in preference across the spectrum than larger numbers. Figure 2 gives the variance in preference across the spectrum for males and females, for Arabic and English samples.

A two-way analysis of variance (ANOVA) with the independent group factors of Gender (Male/female) and Culture (Arabic/English) was conducted on the variance scores to investigate differences in how much variation there is in preference across the spectrum for the different sub-groups. There was a significant difference in the amount of variation in the preference curve of Arabic (mean = 1004.54, SD = 264.91) and English (mean = 903.46, SD = 286.76) samples, *F*(1,112) = 9.43, MSE = 48563, *p* < 0.005. There was also more variation in preference across the spectrum for females (mean = 994.51, SD = 201.30) than males (mean = 919.25, SD = 264.91), *F*(1,112) = 6.55, MSE = 48563, *p* < 0.05. There was also a significant interaction of Culture and Gender, *F*(1,112) = 5.35, MSE = 48563. The ANOVA was followed by independent *t*-tests (two-tailed) to further investigate the reason for the interaction. For English, there was a significant difference between males and females, *t*(24.2) = 2.27, *p* < 0.01, but there was no gender difference for the Arabic sample, *t*(66) = 25, *p* = 0.81.

## DISCUSSION

The main aim of this study was to investigate color preference, by replicating Ling and Hurlbert (2007) and Hurlbert and Ling’s (2007) study but with Arabic participants. The study aimed to answer several questions. First, are there cultural differences in the shape of the overall preference curve for English and Arabic participants? Second, does the sex difference in color preference that was previously found for English and Chinese samples extend to an Arabic sample? If so, this would be support for Hurlbert and Ling’s claims that the sex difference in the weighting of this biological component is universal and possibly linked to evolutionary processes. It was hoped that addressing these questions would contribute to debates about how color preference is constrained, whilst also testing whether a model which summarizes color preference in terms of biological constructs can successfully explain variation in color preference for cultures other than English and Chinese.

### DIFFERENCES IN HUE PREFERENCE ACROSS THE SPECTRUM FOR ENGLISH AND ARABIC

The results indicate that there are differences in hue preference for Arabic and English samples. Collapsing across males and females, the preference curves for the Arabic and English samples have peaks in different locations. For the Arabic sample, preference is highest for the reddish hues, whilst for the English sample, preference is highest for the blue-green region. There are also striking similarities in the preference curves of the two groups. In the green and green-yellow region, the preference ratings are almost identical for Arabic and English, with preference dropping at green-yellow. When males and females are considered separately, it becomes clear that Arabic and English males are more similar in their preference than Arabic and English females. The preference curve for Arabic and English males is highly similar, yet the shape of the curve is dissimilar for Arabic and English females. Arabic females have a strong preference peak for two of the reddish hues—other colors were either at 50% (no preference) or, in the case of greenish hues, were below 50% (an aversion to the color) on the preference scale. English females on the other hand had a preference curve that had minima at green-yellow and yellow-red, but that gradually peaked in between these hues at purple to blue-green. There therefore appears to be a cultural difference in the color preference of Arabic and English females.

The Arabic sample overall also had stronger preferences than the English sample as there was more variation in their preference curve than the English sample. The weaker variation in the preference curve for the English sample compared to the Arabic sample, was due to less variation for English males than females. Therefore, English males appear to be less strong in their hue preferences than English females, yet this sex difference is not found for the Arabic sample.

### IMPLICATIONS FOR THEORIES OF COLOR PREFERENCE

The current study provides evidence for both sex differences and culturl differences in hue preference. As outlined in the introduction, there is a long history of research that has aimed to establish whether color preference varies across different groups and whether there are any universal patterns of color preference. Some of the findings of the current study appear similar to previous color preference studies. For example, McManus et al. (1981) found that females show less preference for yellow than males, and here we also find less preference for yellowish hues for females than males. However, comparisons across color preference studies that have simply summarized color preference using the subjective names for color are difficult to make as colors with the same color term can vary dramatically. The approach used in the Ling and Hurlbert studies summarizes color preference in terms of how stimulus-background cone-contrast is weighted summarizing color preference quantitatively rather than using subjective color names. Hurlbert and Ling (2007) and Ling and Hurlbert (2007) argue that this approach is better as color preference is identified in terms of biologically meaningful constructs (the cone-opponent mechanisms) whilst also quantifying how well these biological components account for variation in preference. They argue that this method enables comparisons across groups (such as by sex and culture) to be made efficiently with reference to the underlying mechanisms of preference. Here it is shown that their approach can be extended to other cultures and we show that Arabic color preference can also be identified in terms of the cone-opponent mechanisms and comparisons between Arabic and English can be made by comparing the weights on these mechanisms.

The findings have implications for theories on color preference. Some have argued that there is a universal order of color preference (e.g., Eysenck, 1941) and others have argued for universal sex differences in color preference (e.g., Ling and Hurlbert (2007)). However, the findings of the current study suggest that color preference varies with both sex and culture and that sex differences in color preference also vary culturally. Studies of color preference need to move away from simply summarizing, quantifying and comparing color preference across cultures, sexes and ages and start to consider the factors that lead to variation. The extent to which color preferences driven by interactions with the chromatic environment, such as colors being associated with positive or negative objects, also needs to be established. For now, the strongest contribution from the current study to the literature on color preference, is that Hurlbert and Ling (2007) and Ling et al.’s (2007) suggestion that there are evolutionarily driven universal sex differences in the weighting of L-M for color preference is not supported.

## CONCLUSION

The current study considered whether there is cultural variation between Arabic and English in color preference. The study used Ling and Hurlbert (2007) and Hurlbert and Ling’s (2007) approach which summarizes color preference in terms of weights on the two cone-opponent processes. A substantial amount of variation in color preference color be explained for both Arabic and English speakers using this approach. Arabic and English preference curves were found to differ, yet there was greater similarity for Arabic and English males than Arabic and English females. There was also a sex difference that was present for both Arabic and English participants. Support for Ling and Hurlbert (2007) and Hurlbert and Ling’s (2007) claim for a universal sex difference, meaning that females universally prefer hues redder than the background, was not provided by the findings.

### Conflict of Interest Statement

The author declares that the research was conducted in the absence of any commercial or financial relationships that could be construed as a potential conflict of interest.
